# “An ox sitting on One's chest”: Experiences and understandings of common mental health conditions of Turkish‐speaking immigrants with lived experience in the UK

**DOI:** 10.1111/papt.70084

**Published:** 2026-06-05

**Authors:** Ayse Akan, Gill Green, Frances Blumenfeld, Danny Taggart, Susan McPherson

**Affiliations:** ^1^ Bogazici University The Faculty of Humanities and Social Sciences, Psychology Department, Translational Clinical Psychology Lab, Boğaziçi Üniversitesi İstanbul Türkiye; ^2^ University of Essex, School of Health and Social Care Colchester UK

**Keywords:** anxiety, causal attributions, clinical psychology, common mental health conditions, culture, depression, explanatory models, help‐seeking attitudes, immigrant, lived experience, qualitative research, reflexive thematic analysis, somatisation, symptom expression, symptom manifestation, Turkish

## Abstract

**Objectives:**

Exploring how Turkish‐speaking immigrants understand and express common mental health conditions is crucial, as discrepancies in this area have real‐life consequences for treatment. Some key concepts to examine within this are the long‐standing belief that Turkish‐speaking immigrants somatise emotional difficulties and cannot identify them, and that they predominantly rely on traditional explanatory models of mental health. Accordingly, this study aimed to explore how this population experiences, expresses and understands these conditions and to examine their coping resources and help‐seeking attitudes.

**Design:**

Qualitative design.

**Methods:**

Semi‐structured interviews were conducted in Turkish with 18 Turkish‐speaking immigrants who self‐identified as having experienced common mental health difficulties. Data were analysed inductively using reflexive thematic analysis within a critical realist framework.

**Results:**

Four themes with ten subthemes were generated: symptom presentation, explanatory models, coping strategies and help‐seeking attitudes. Participants reported both emotional and physical symptoms, often expressed through rich idioms and metaphors. They attributed their distress to upbringing, traumatic events, chronic adversities, discrimination, interpersonal relationships and their own perceived shortcomings. Coping included personal, interpersonal, community and religious resources alongside lay resources. While most participants were open to professional help, all expressed distrust towards UK services and many preferred Turkish‐speaking professionals.

**Conclusions:**

Contrary to popular belief, Turkish‐speaking immigrants articulated distress in both emotional and physical terms. Their explanatory model was mainly psychosocial but also included biological and traditional elements, reflecting a dynamic, multi‐model approach. Moving beyond reductive stereotypes about Turkish‐speaking immigrants and Global South populations is crucial for providing meaningful and effective care.

## INTRODUCTION

Common Mental Health Conditions^1^ (CMHCs), comprising depression, anxiety and related disorders (NHS England, 2025), contribute significantly to the global burden of disease (Walker et al., 2015; Whiteford et al., 2015). Studies suggest that immigration can play a role in increasing vulnerability to developing mental health challenges, including CMHCs (Bhugra & Jones, 2001; Kirmayer et al., 2011). Since migration is a dynamic process involving acculturation and adaptation, it can increase vulnerability to these conditions (Kirmayer et al., 2011). This process often leads to feelings of alienation, contributing to the development of CMHCs among immigrant populations (Bhugra, 2004; Zhong et al., 2018).

In England, CMHCs affect up to 20.2% of the adult population (NHS England, 2025). While ethnic minority groups often report higher rates of mental health conditions than White British individuals (McManus et al., 2016), they remain significantly under‐represented in primary care services (McKenzie, 2008). This may suggest a disparity in help‐seeking and service access that warrants closer investigation (Eylem et al., 2016).

This vulnerability is particularly evident among Turkish‐speaking migrants, who frequently experience higher rates of CMHCs compared to both native populations and other immigrant groups (Sempértegui et al., 2023). Extensive research across Europe, including Austria (Strohmeier & Dogan, 2012), Belgium (Beirens & Fontaine, 2011; Levecque et al., 2007), Germany (Beutel et al., 2016; Janssen‐Kallenberg et al., 2017; Makowski & von dem Knesebeck, 2017; Reich et al., 2015; Sariaslan et al., 2014; Schouler‐Ocak et al., 2008; Tydecks et al., 2009), the Netherlands (Bengi‐Arslan et al., 2002; Fassaert et al., 2009; Knipscheer & Kleber, 2005; Schrier et al., 2010; van Bergen et al., 2010; Velthorst et al., 2012) and the UK (Britton et al., 2000), supports this trend.

Additionally, studies have shown that Turkish‐speaking immigrants report higher levels of depressive and anxiety difficulties when compared to other immigrant groups in host nations. More specifically, in the Netherlands, Turkish speakers reported higher levels of distress compared to Polish immigrants (Beutel et al., 2016), in Norway, compared to Iranian, Pakistani, Sri Lankan and Vietnamese immigrant groups (Lien et al., 2010), and in Australia, compared to Vietnamese and Filipino immigrants (Small et al., 2003). Notably, distress levels seemed to vary by host country as well; for instance, Turkish immigrants in Norway exhibited higher distress than those residing in Sweden (Virta et al., 2004).

Despite these high rates of emotional difficulties, there has been very little research on how Turkish immigrants experience, understand and cope with these mental health challenges. The limited research available has usually been conflated with ‘somatisation theory’. This theory, also known as the ‘somatisation hypothesis’, is a long‐standing belief in the literature that originated around 40 years ago and is still held by some researchers and healthcare professionals today. Proponents of this view believe that people from the Global South,^2^ including Turkish‐speakers, have greater difficulty identifying and expressing their emotions and express emotional difficulties through physical symptoms (Angel & Thoits, 1987; Arnault, 2009; Balkir, 2013; Bragazzi et al., 2014; de Bruyn, 1989; Kleinman, 1977; Leff, 1988; Moodley et al., 2018; Nakkas et al., 2019).

Furthermore, some suggest that people from the Global South, including Turkish‐speakers, attribute psychological problems to traditional or spiritual causes more frequently than those in the Global North (Balkir, 2013; de Bruyn, 1989; Ghane et al., 2010; Nickel et al., 2006; Ozturk & Volkan, 1971; Reich et al., 2015). Consequently, the resources people of the Global South employ to cope with mental health conditions were thought to consist primarily of traditional or spiritual practices. Therefore, it has been suggested that populations in the Global South are unsuitable for talking therapies, as they are supposedly unable to identify or access emotions. Additionally, it is assumed that if individuals attribute their mental health conditions to traditional or spiritual causes, they would seek help from a traditional/spiritual leader rather than healthcare professionals.

However, a recent systematic review challenged this explanation and indicated that Turkish‐speaking immigrants do identify and communicate their emotions and use a psychosocial model of understanding mental health conditions with some traditional, spiritual and medical elements (Akan et al., 2023). This, alongside another systematic review (Sempértegui et al., 2019), which included research conducted with Turkish‐speaking populations in Europe where no UK study was found eligible for the study, highlighted that while Turkish‐speaking immigrants had more somatic complaints of CMHCs than host populations (and at times other immigrant groups), they also consistently had higher rates of emotional difficulties in comparison to host and other immigrant populations.

It is estimated that there are 180,000–250,000 Turkish‐speaking immigrants living in the UK (Sirkeci & Esipova, 2013), and the total estimate of Turkish‐speaking immigrants is considered to be six and a half million worldwide (Republic of Türkiye Ministry of Foreign Affairs, n.d.). Simply labelling all of these populations with somatisation and traditional causal attributions, and assuming they will not seek help because of these factors, can limit access to direly needed treatment or prevent services from being appropriately tailored to their needs. Therefore, it is crucial to understand the actual experiences and understandings of British Turkish immigrants in order to offer them the best possible care. Given that Turkish‐speaking immigrants are consistently found to have more mental health difficulties than natives and other immigrant populations, it is important to gain an understanding of their experiences of CMHCs. The limited and controversial information on their struggles might result in Turkish‐speaking people having inadequate or no access to mental health services; furthermore, psychologising physical experiences may also have a detrimental impact on their access to physical healthcare as well (Tan et al., 2020).

For this reason, the present research aimed to investigate how Turkish‐speaking immigrants in the UK experience, express and understand their own CMHCs as well as the resources they employ to cope with CMHCs and their attitudes to seek help for CMHCs.

## METHODS

### Design and data analysis

The study adopted a qualitative design, and the acquired data were analysed using Reflexive Thematic Analysis (RTA) (Braun & Clarke, 2022) with a critical realist philosophical positioning (Larkin et al., 2006), utilising the help of the computer software package NVivo version 12. All interviews were voice‐recorded with the interviewees' permission, and transcriptions were prepared verbatim in Turkish by the first author. Coding and analysis were also conducted in Turkish, and selected quotations were translated into English by the first author, who is a native Turkish speaker with professional‐level English proficiency. Discrepancies in the clarity of specific quotations were resolved through consultation with the second and third authors, whose first language is English. The first author subsequently integrated this collective feedback to refine the final selections.

The analysis adopted an inductive approach, meaning the data were used to develop the construction of the analysis without a pre‐existing coding frame. The research was closely linked to the manifest meaning approach, as it enquired about explicit experiences, but the first author also worked on implicit/latent content to an extent to explore the participants' understandings. During the second stage of analysis, initial codes were derived from the most basic elements of the data according to the core content. The first author took both manifest and latent meaning into consideration and attempted to label the codes accordingly by constantly considering the differences between the participants' and the researcher's points of view.

A second independent coder was not involved in the coding process. Practically, because the language of the interviews was Turkish, the dual coding of the data with the non‐Turkish‐speaking supervisory team was not applicable. Furthermore, this decision aligns with the theoretical principles of RTA (Braun & Clarke, 2022), which conceptualises coding as an inherently subjective and interpretive practice rather than a process requiring a search for objective consensus. To ensure study rigour and maintain reflexivity, the first author kept a log and a reflective journal before and after each interview and took extensive notes while coding the data and working on meanings. The wider research team provided guidance, and potential themes and sub‐themes were reviewed, combined, split or refined after discussion with the co‐authors.

### Instruments

The first author conducted semi‐structured interviews utilising an open topic guide, and participants completed a demographic information sheet. The author adopted a humanistic approach, as she was primarily interested in the participants' perceptions (Plummer, 2001), viewing the interviews as “conversations with a purpose” (Burgess, 1990, p. 84) as reported by Mason (2002). The coding and thematic maps were conducted using NVivo software version 12.

### Participants

Following principles of RTA, sample size was determined in advance based on the scale of the research, and eighteen participants were recruited for this medium‐sized study (Braun & Clarke, 2013).

Inclusion criteria: Turkish‐speaking persons who (1) were originally from Türkiye or had a parent who migrated from Türkiye; (2) had been a documented permanent resident in the UK, hence eligible to register with a GP practice; (3) had identified themselves as having or having had emotional difficulties; (4) had not received talking therapies for their emotional difficulties (to focus on the understandings and experiences of lived emotional difficulties rather than therapy processes); and (5) were over eighteen at recruitment.

Purposeful sampling techniques (intensity sampling and snowball sampling) were adopted (Suri, 2011), while an effort was made to include a diverse sample, such as men and people who live outside big cities in the UK. An advertisement leaflet using a non‐medical language, and instead including common phrases and metaphors in the Turkish language used to describe emotional distress, was prepared by the researcher and shared on social media groups aimed at Turkish‐speaking people, including WhatsApp and Facebook.

### Data generation

People who volunteered for the study were recruited after establishing their eligibility and obtaining verbal consent. Fourteen one‐to‐one interviews, and for practical reasons, one interview with four participants, were conducted in a group setting. The participants were interviewed in confidential but public spaces (*n* = 9), on WhatsApp Video Call (*n* = 3) or in their homes (*n* = 6). The interviews ranged in length from 35 to 100 min (*M* = 70 min).

Before the interviews, participants were asked to read the information sheet, sign an informed consent form and complete a demographic questionnaire. All communication and interviews were conducted in Turkish by the first author, a native Turkish speaker. The interviews were conducted following ethical guidelines for the conduct of research with human subjects of the University of Essex and the British Psychological Society (BPS).

### Ethics

Ethical approval from the University of Essex was obtained, and the university's and BPS's guidelines were followed. Informed consent was sought both verbally and in writing from all participants in Turkish. The data were anonymised, and participants were assigned pseudonyms.

No participants expressed any emotional distress during the interviews. Post‐research support was offered, and a participant who reached out post‐interview for an issue with regards to a family member was signposted to a relevant service.

### Reflexivity

Researchers positioning themselves outside the researched material is deemed impossible by some, since they will inevitably have a relationship with the phenomena under investigation (Willig, 2013). The first author (who carried out interviews and led the analysis) considers herself to be a ‘partial insider researcher’ since she met all the inclusion criteria except for being a permanent resident in the UK. She adopts a biopsychosocial understanding of mental health, with an emphasis on psychosocial, and her values align closely with critical mental health and anti‐racism perspectives. Despite this, she acknowledges some internalised stereotypical views regarding the mental health of Turkish‐speaking people, mainly assuming that Turkish‐speaking people express their emotions by somatisation prior to this study. The other authors, who guided and advised on the research process, are academics based permanently in the UK, with a mixture of backgrounds including medical sociology, social psychology and clinical psychology. Two are also registered clinical psychologists with practitioner status in the UK. All had some previous experience of researching mental health understandings of different cultural and ethnic groups, but generally had no specific preconceived ideas of how Turkish‐speaking migrants might have different understandings of mental health compared to other ethnic minority groups in the UK.

To maintain a reflexive approach, the first author kept a log and a reflective journal before and after each interview and reflected on interviews during the data collection phase with the fourth author. She also took notes while coding the data and working on manifest and latent meanings. Furthermore, the first author consistently checked her interpretations to see whether she was leaning towards interpretations aligned with her own values without evidence. For example, when a participant mentioned during an interview how she was against being labelled as ‘depressed’, the author double‐checked with the participant to see whether the participant was against the construct of ‘depression’. By doing so, she learnt that the participant was not against the construct per se; rather, she was expressing her disapproval and frustration over how this diagnosis was delivered to her by a 111 operator via a computer system, without a doctor seeing her. All authors provided feedback, guidance and supervision on the analytic process at different stages.

## RESULTS

### Demographics

The participants were aged between 25 and 57 (*M* = 36). The mean time spent in formal education was 12.22 years. Of the participants eleven identified as Turkish, seven identified as Kurdish or Zaza, while some also claimed different identities such as British or *Türkiyeli*.^3^ Table 1 below summarises other demographic information about the sample, and Table 2 offers an overview of the participants with pseudonyms.

**TABLE 1:** Demographics.

		Number (percent)
Gender	Female	16 (88.9%)
	Male	2 (11.1%)
Highest Education	Primary	1 (5.6%)
	Middle	4 (22.2%)
	High‐School	5 (27.8%)
	Associate's*	3 (16.7%)
	Bachelor's	4 (22.2%)
	Master's	1 (5.6%)
Immigration Route	Ankara Agreement Visa	3 (16.7%)
	Spouse Visa	10 (55.6%)
	Asylum	4 (22.2%)
	Family Reunion	1 (5.6%)
Economic Status	Low (with benefits)	3 (16.7%)
	Middle (with benefits)	6 (33.3%)
	Middle (no benefits)	8 (44.4%)
	High	1 (5.6%)
Marital Status	Single	2 (11.1%)
	Married	16 (88.9%)
Children	Yes	14 (77.8%)
	No	4 (22.2%)
Identity***	Turkish	7 (38.9%)
	Turkish and Türkiyeli**	2 (11.1%)
	Turkish and British	2 (11.1%)
	Kurdish and Türkiyeli	2 (11.1%)
	Kurdish and Türkiyeli and British	2 (11.1)
	Zaza and Türkiyeli	1 (5.6%)
	Zaza and Türkiyeli and British	1 (5.6%)
	Turkish and Kurdish and Türkiyeli and Human	1 (5.6%)
Residence	London	9 (50%)
	North East	6 (33.3%)
	South West	2 (11.1%)
	West Midlands	1 (5.6%)

* Associate's degree is a two‐year university degree, which can be studied following high‐school graduation. It is generally accepted as a pre‐bachelor foundation degree by UK universities.

** Türkiyeli means ‘from Türkiye’ and is generally used by non‐Turkic populations from Türkiye.

*** Some participants added other identities of their own choice into the list.

**TABLE 2:** Participant Pseudonyms with Selected Demographics.*

Pseudonym	Sex	Age	Latest degree
Serpil	Female	Late‐twenties	Associate's
Güneş	Female	Mid‐twenties	Master's
Elif	Female	Late‐twenties	High School
Zehra	Female	Early‐thirties	Middle School
Hilal	Female	Late‐twenties	Bachelor's
Hacer	Female	Mid‐thirties	Associate's
Zeynep	Female	Late‐thirties	Bachelor's
Neriman	Female	Mid‐forties	Middle School
Şerife	Female	Mid‐forties	Primary
Ayten	Female	Early‐forties	Associate's
Seher	Female	Mid‐twenties	Middle School
Zuhal	Female	Mid‐thirties	Middle School
Meryem	Female	Early‐forties	High School
Melike	Female	Mid‐thirties	High School
Yeşim	Female	Early‐thirties	High School
Mesude	Female	Early‐forties	Bachelor's
Orhan	Male	Early‐forties	Bachelor's
Ali	Male	Late‐fifties	High School

* Associate's degree is a two‐year university degree, which can be studied following high‐school graduation. It is generally accepted as a pre‐bachelor foundation degree by UK universities.

### Thematic analysis

Table 3 gives a summary of the themes and sub‐themes. Four themes were constructed, incorporating ten sub‐themes. Theme and sub‐theme distribution among the sample can be examined in Table 4.

**TABLE 3:** Themes and Sub‐Themes.

Themes	Sub‐themes
1. Souls in Distress and Bodies in Pain	
2. What Happened to Me and What I Did to Myself	2a. Whispers from childhood
	2b. Things you can't recover from that easily
	2c. Diminished lives
	2d. Feeling like an outsider
	2e. Hurt by dearest
	2 f. “I did this to myself”
3. “Going to Doctors isn't the Only Cure”	3a. Leaning on others
	3b. Drinking tea and watching emotional films
	3c. Pouring grief into Allah
	3d. Being stronger while knowing it isn't always possible
4. Keeping Silent in front of Professionals	

**TABLE 4:** Theme and sub‐theme Distribution among Participants*.

Participants	Serpil	Güneş	Elif	Zehra	Hilal	Hacer	Zeynep	Neriman	Şerife	Ayten	Seher	Zuhal	Orhan	Ali	Meryem	Melike	Yeşim	Mesude
Souls in Distress and Bodies in Pain																		
	✔	✔	✔	✔	✔	✔	✔	✔	✔	✔	✔	✔	✔	✔	✔	✔	✔	✔
What Happened to Me and What I Did to Myself																		
Whispers from childhood	✔	✗	✗	✗	✔	✔	✔	✔	✗	✗	✗	✔	✔	✗	✗	✗	✗	✗
Things you can't recover from that easily	✔	✔	✔	✗	✔	✗	✗	✔	✔	✗	✔	✔	✗	✔	✗	✗	✗	✗
Diminished lives	✔	✔	✔	✔	✔	✔	✔	✔	✔	✔	✔	✔	✔	✔	✔	✔	✔	✔
Feeling like an outsider	✔	✗	✔	✔	✗	✔	✔	✔	✔	✔	✔	✗	✔	✔	✗	✗	✔	✔
Hurt by dearest	✔	✔	✔	✔	✔	✔	✔	✔	✔	✔	✔	✔	✔	✔	✗	✔	✔	✔
“I did this to myself”	✔	✔	✔	✔	✔	✔	✔	✔	✔	✔	✔	✔	✔	✔	✗	✔	✔	✔
‘Going to Doctors isn't the Only Cure’																		
Leaning on others	✔	✔	✔	✔	✔	✔	✔	✔	✔	✔	✔	✔	✔	✔	✔	✔	✔	✔
Drinking tea and watching emotional films	✔	✔	✗	✗	✔	✔	✔	✔	✗	✔	✔	✔	✔	✔	✗	✗	✔	✔
Pouring grief into Allah	✗	✗	✔	✔	✔	✔	✗	✔	✗	✔	✗	✔	✗	✔	✔	✔	✗	✗
Being stronger while knowing it isn't always possible	✗	✗	✗	✔	✗	✔	✔	✔	✔	✔	✗	✔	✔	✔	✗	✗	✔	✗
Keeping Silent in front of Professionals																		
	✔	✔	✔	✗	✔	✔	✔	✔	✔	✔	✔	✔	✔	✗	✗	✔	✔	✔

* The Last four participants (Meryem, Melike, Yeşim and Mesude) were interviewed together in a group setting in a shorter amount of time (total 35 min). This might have had an impact on their contributions.

#### Theme 1. Souls in distress and bodies in pain

This theme incorporates how distress manifested itself in the participants' experiences. Both psychological and bodily experiences were discussed, and links between the two were made by some. Medical language was used scarcely, but when talking about a behaviour which they found excessive, some used the term “illness” (“cleaning illness”, “symmetry illness”). A small number of phrases or idioms which are rooted in bodily experiences but convey a psychological meaning were also used by the participants: e.g., ‘nerves’ (*sinir‐ler*).

All of the participants used rich, colourful and extensive language to describe psychological distress in Turkish. The Turkish word *sıkıntı* (Akan et al., 2023), which refers to a general state of feeling psychologically unwell, or a sense of dread, was often used by participants. As it refers to a general state rather than a specific condition, individual experiences of *sıkıntı* varied. In this study, *sıkıntı* is translated as ‘distress’. Additionally, participants talked about feeling “low”, “sad” and “depressed”. They also talked about lacking “enthusiasm”, “desire”, “motivation”, “pleasure” or a “desire to live” as well as feeling “purposeless” and “empty”.

Feeling “hopeless” and “desperate”, “feelings of worthlessness”, “excessive crying” and “feelings of guilt” were mentioned. Many also talked about “having less tolerance”, and in return, “getting hurt easily”, “getting irritated easily”, or “getting angry easily” to the point that they have “anger outbursts”.

Anxiety‐related experiences such as “feeling worried”, “scared” and “panicky” were raised. “Having groundless fears” was also discussed. Some participants talked about having “intrusive thoughts”, being “fixated on things” or “having obsessions” and “picturing unlikely things” or “creating disaster scenarios in one's head”. Some participants talked about behaviours which could be labelled as compulsions, such as locking and unlocking one's car's doors repeatedly.

Excessive cleaning, “cleaning illness”, “being too meticulous” and “not being able to be flexible in doing things” were also talked about by a small number of participants.

The majority of the participants also used words or phrases to describe how psychological distress manifested itself on their bodies. When talking about their physical distress, the participants used common but also some not‐so‐common phrases and metaphors in Turkish.

The physical presentations described can be grouped into different domains. First, feeling pain in the body, such as “stomach‐aches” or “headaches”, was stated. Muscles or body parts becoming tense or tightened was another feeling mentioned: “stiff shoulders” and “tension” in the body were raised:

*My nerves were wrecked for a while, I was waking up with shoulders like this* [tenses her shoulders] *due to nerves, it started to affect my body, I was feeling very dizzy’*.Zuhal.

Presentations which could be related to hyperventilating and shortness of breath were expressed, some using straightforward language but others using phrases containing more personal experience. For instance, *Meryem* used an uncommon expression, “bones and heart getting entangling with each other” to further explain her heart palpitations.

Cognitive and/or neurological difficulties such as “forgetfulness” and “sleep difficulties” were raised. Additionally, fatigue was experienced by some participants. *Zuhal* described finding getting up from bed really difficult by saying “It is as if I am nailed to the bed”, whereas *Elif* used the term “becoming lifeless”. Another colourful metaphor used was “having an ox residing on one's chest”:

*But you know what*, [*as if*] *something fell on me, an ox completely sat on me* [laughs] (*…*) *It was as if you get paralysed. I mean I have so many thoughts in my head, so many thoughts in my head, emotions, feelings, things I dream of …* [*but*] *my hands and arms don't move*. Hilal.

#### Theme 2. What happened to me and what I did to myself

This theme incorporated participants' understandings of the causes of their distress. These explanations were mainly psychosocial in nature; participants thought that they had developed common mental health difficulties due to adverse life circumstances. At the same time, some had biological (season change, weather, alcohol, and menstruation) and/or traditional explanations (*nazar*). Nevertheless, all the participants made links between their distress and social circumstances. These are discussed in detail in the following sub‐themes.

**Subtheme 2a** **: Whispers from Childhood**. Nearly half of the participants commented on their upbringing and early life circumstances as an explanation for their distress. Religious education, societal expectations and early schooling experiences were also discussed.

Participants reported that their parents asked too much of them when they were little:

*I am angry at how my mother brought me up, she gave me too many responsibilities … I took on too many responsibilities, there was so much to do*. Serpil.

On the other hand, some parents gave too few responsibilities to their children, consequently making their children too dependent on them.

*They* [*parents*] *always brought us up dependent on themselves. I didn't think I could cope on my own, I didn't have this concept. This is why I felt so desperate at that time*. Zuhal.

*Hacer* thought that a combination of her parents' upbringing and the religious education, which she had received had affected her decisions in later in her life:

*Maybe it is something our parents whispered into our ears when we were little, or maybe … I don't know … we got religious education* (*…*) [*I thought*] *I need to put myself in God's hands, I will accept this*. Hacer.

**Subtheme 2b** **: Things You Can't Recover from That Easily**. Some, but not all, participants talked about traumatic incidents which had caused major distress for them and affected their wellbeing. These incidents included: nearly fatal illnesses and accidents in the family, separation in the family, death in the family, domestic violence and traumatic asylum‐seeking experiences.

For instance, *Neriman* had to leave her then three‐year‐old daughter in Türkiye during the asylum‐seeking process, thinking that she would join her soon, but unfortunately, it took three years for the family to be reunited:

*I felt so empty, so sad, became very unwell with headaches* (*…*) *my child was just three years old* (*…*) *after my daughter arrived here, I started to feel better* (*…*) *but I always carry that on my conscience*. Neriman.

**Subtheme 2c** **: Diminished Lives**. The participants discussed adversities that were not major stressors, but living with them for a long time built up a cumulative effect and influenced their mental wellbeing. The principal concepts were the harmful effects of being away from wider family, fewer opportunities for socialising due to scarcity of alcohol free spaces, distress caused by everyday struggles, including language problems, and unmet expectations.

Being deprived of the support of wider family and community was mentioned by *Mesude, w*ho initially lived in a small UK town with very few Turkish‐speaking people.

*I realised that having someone around, even when you can't tell them what is going on, is a source of support. People in Türkiye have that, but you have nobody here*. Mesude.

Many participants made comments on the lack of appropriate activities for them. This came through especially strongly from people who lived outside London:

*There is nowhere to go, nothing to do* [*here*]. Güneş.

Other struggles included the citizenship test and language problems. Half of the sample had limited English skills and encountered various difficulties because of that. *Hilal* explained that after her father's business went bankrupt, she had to take care of benefits applications with limited English skills:

*We had been asking for an interpreter but they didn't send us one from the job centre* (*…*) *I was very distressed because of that* (*…*) *I started to have ‘symmetry illness’, wanting everything grouped in a particular order and colour*. Hilal.

Unmet expectations or unexpected repercussions were other concepts that came across. Participants had disappointments related to how their lives had turned out, which contributed to their distress. These had a wide range from financial to personal issues:

*I had a desk job back in Türkiye* (*…*) *Then you end up here and find yourself washing dishes in a café kitchen*. Mesude.

On top of difficulties from being an immigrant as detailed above, participants also mentioned the demanding nature of other circumstances, which could be relevant to any British resident, such as financial and academic problems.

**Subtheme 2d** **: Feeling Like an Outsider**. Participants described experiences of racism, discrimination and exclusion and feelings of alienation and acknowledged their contributory effect on their emotional wellbeing.

While the experience of feeling like an outsider was common across the sample regardless of their worldviews, the contexts in which they experienced this alienation differed according to their ethno‐cultural and religious backgrounds. For instance, visibly Muslim women wearing headscarves frequently reported being the direct targets of overt physical and verbal abuse. Conversely, participants who were not visibly Muslim or those from more secular backgrounds, did not report overt racist attacks; instead, they experienced alienation through more subtle prejudices or profound cultural and communicative disconnects with the host society.

*Zehra* is a practising Muslim woman who wears a headscarf and lived in a small city in the South West of England. She talked openly about being a target of physical and verbal abuse, which was sometimes carried out by children. *Zehra* had witnessed some families encouraging their children to attack ethnic minorities, knowing that the victim would not be able to retaliate against a child. *Zehra* had tried to ask for help from the police but was not satisfied with the outcome:

*I went to the police a couple of times, but even they … For example, they said maybe they showed you a number* [referring to a rude hand gesture], *maybe they didn't mean it, it is a child … They [the police] even tried to defend them. So, I went to the police once, twice … but didn't want to go for a third time*. Zehra.

Negotiating what they were told or thought about the UK, what they had experienced in the UK, and what they had brought with them from Türkiye was constantly evaluated by the participants while they were trying to make sense of their experiences. Upon their arrival in the UK, the majority of participants heard stories from other Turkish speakers about physical and verbal attacks on Muslims or ethnic minorities by racists. *Zeynep*, who also wears a headscarf, was among those warned of racism when she moved to a small city in the East Midlands. She explained that she had moved there “in fear and with prejudice”. However, when she settled there, she slowly created a way to exist in that environment.

On the other hand, as a man of a more secular background, *Orhan* did not report overt racist attacks against him. However, he did talk about how the Turkish and British styles of communication differed and how it was hard to meet on common ground with British people:

*The English are very decent, helpful, charitable, well the* [*English*] *people I know generally are, but they keep you at arm's length, they don't actually let you in*.(*…*) *You can't form deep bonds with that person*. Orhan.

Even though they differed from those of other participants, *Orhan* also had his own preconceptions about the UK and British people before his arrival in the UK. From a young age, *Orhan* had wanted to emigrate to a Western country because he perceived Western people to have an “open‐minded world view” and that they knew how to be “happy”. He hoped that if he was to live in a Western country, he would also start enjoying life. However, to his dismay, it did not happen:

*I really don't know* [*why it didn't happen*], *I guess because that wasn't the case* [laughs]. *Because this is not something that is transferred, I mean, when you come here you don't get software* [*which can be*] *downloaded; it is something in your head in the end. Meaning, I guess it is not just outside factors but also the inside ones*. Orhan.

**Subtheme 2e** **: Hurt by Dearest**. All of the participants talked about distressing experiences related to family and those close to them. Additionally, relationships with other family members, friends and other Turkish‐speaking people in the communities were raised.

In this context, factionalism within the Turkish‐speaking community was brought up. Many put forward the idea that being a fellow Turkish‐speaker did not guarantee a harmonious friendship, as people from different ethno‐cultural and religious groups have different lifestyles and values. For instance, sectarian (*mezhep*) differences, such as those between Sunni and Alevi communities, were introduced as a challenge to building or maintaining good relationships. *Güneş*, a Sunni participant, expressed frustration over feeling that her religious practices were not equally respected by her Alevi acquaintances.

*They need to respect, we do that*. (*…*)*You need to respect. I respect you, you also need respect me* [*in return*]. Güneş.

Furthermore, hostile secularism was also cited as a significant source of distress. Participants who wore headscarves for religious reasons discussed the lack of respect which they encountered from Turkish speakers who had a hostile attitude towards the Islamic way of living. *Neriman* reported an incident that happened with a Turkish‐speaking teacher who taught English. According to *Neriman*, this person constantly criticised her for wearing a *çarşaf* (a traditional Islamic over‐garment) and advised her to “be modern”. It sounded as if Neriman felt discriminated against, hurt and possibly betrayed because this prejudice came from one of her “own people”. In the end, *Neriman* dropped out of that course to not to face the disapproval anymore.

**Subtheme 2f** **: “I Did This to Myself”**. In addition to “what happened to them”, many participants talked about their own shortcomings in relation to their mental health, judging their abilities, behaviours, and selves to contribute to their distress. Some participants thought they exaggerated their difficulties, as others in similar situations were not as affected as them:

*I guess we exaggerate things a little bit too much, well … drowning ourselves in sorrows, complaining about things …* Hacer.

Another point was made about feelings of inadequacy and being weak. Some participants conveyed how they thought that they were not able to do what was necessary to help themselves:

*I can't make myself happy because I am not happy with the work I do* (*…*) *it is as if I am rowing against the tide, like a cat chasing its tail … there is always a storm in my head … I am doing something very wrong but I don't know exactly what*. Ayten.

A further point was the behaviour or actions which they took or sometimes did not take. Self‐blame was rooted in all of the concepts which were raised.

*It is always you who you blame. In fact, people always blame themselves, even when they seem like they are blaming someone else, they always blame themselves*. Mesude.

Finally, harmful actions against the self were also raised. This included “misdirecting oneself”, “sabotaging oneself”, “punishing oneself”, “putting pressure on oneself” or “drowning oneself in sorrows”. *Şerife* also talked about meta‐distress; how by getting distressed over things she thinks she should not fixate on made her even more distressed:

*Well, I did this to myself. I fixated on these things and that* [the fixation] *harmed me … It wasn't something I could control* [at that time]*; of course you regret it afterwards*. Şerife.

#### Theme 3. “Going to Doctors isn't the Only Cure”

All of the participants acknowledged using particular ways to cope with distress. Some who did not find their strategies particularly helpful were searching for more effective ways, whereas others thought that their ways were best for themselves, and did not think they needed anything else. Additionally, some participants underlined the limited value of these strategies, stating that their distress lifted only after there was a change in their circumstances. Four sub‐themes were generated in relation to this theme, which can be found below.

**Subtheme 3a** **: Leaning on Others**. Participants talked about feeling better thanks to the help of close ones. The main source of human support was from the immediate family: spouse, siblings and sometimes parents, even though some said that they had reservations about sharing their distress with parents in order not to worry them.

*We help each other, [going to] doctors isn't the only cure, you can get help from people around you. When there are people who understand you, they do things for you, they do it for you, and then you do it for them. This is a remedy as well*. Zehra.

Some participants talked about the importance of friends for getting practical and emotional support. *For instance, Zuhal* talked about the impact of the compassion that was offered without anything being expected in return. At that time, *Zuhal* was staying in a women's shelter following domestic abuse:

*One of my friends, a friend I met at the mosque, invited me to her house a couple of times*. (*…*) *It did me so much good*. (*…*) *I felt like I wasn't alone. Secondly, I started to think that there are still good people out there, because I was thinking there weren't any good people out there anymore*. Zuhal.

Some talked about a wider community support; sometimes a group of people who attended mosques, sometimes more secular organisations, such as a social media group.

Some participants also stated that community work, such as volunteering or teaching, helped distract themselves from their own difficulties. On the other hand, some stated “cutting toxic people out of their lives” as another resource.

**Subtheme 3b** **: Drinking Tea and Watching Emotional Films**. Many talked about activities which they did to keep themselves busy and channel their emotional energy. Reading, attending courses, going back to work, sleeping, watching fiction, ironing, and doing sports were mentioned. *Ali* talked about crying over emotional films:

*I put two kettles on, brew tea, and watch films all night till morning. Will you believe me if I say that I would shed tears, maybe enough to fill those kettles?* Ali.

Crying and drinking tea were cited by others as well. However, some also considered crying as something which happens when they are distressed, not as a coping strategy. Similarly, strategies which might come across as harmful to some, such as avoidance or smoking, were brought up as helpful tools by others.

On the other hand, *Hilal*, who thought that her troubles might be caused by *nazar*, talked about using some traditional methods, such as washing herself with water with vinegar in it, and cupping.

**Subtheme 3c** **: Pouring Grief into Allah**. Many, but not all, talked about how their faith helped them to get through hard times. Sometimes, however, they felt that it did not give them the resilience they hoped for, and they found this surprising.

Even so, many talked about their Islamic faith being a great support for them. Some talked about taking up *salah* and / or being consistent with *salah*.^4^

*It feels like I am having a heart‐to‐heart conversation with Allah, as if I am pouring my grief into him*. Ayten.

Many also mentioned reciting verbal prayers such as *tefriciye* or *esma*, which gave them strength as it created a refuge in Allah. They also thought that even difficulties are sent by Allah, so they accepted them. Stemming from these religious thoughts, some participants stated that they had started to reflect on their life from a different perspective. Many others referred to concepts often associated with Islam, *sabır*, *şükür* and *tevekkül*.

*Sabır* means being resilient in the face of adversity:

*I didn't have* sabır *in the past, I learnt how to do that. I am learning it through big tests*. Elif.

*şükür* means being grateful for whatever you already have:

*That actually relaxes me a little bit, you learn* şükür *in a sense*. (*…*) *Since we are away* [from Türkiye], *we really miss that environment and realise how valuable it is*. Melike.

and *tevekkül* means doing everything you can do avoid negative outcomes but also knowing that everything is in the hands of Allah, and therefore seeing Allah in charge and not bothering yourself with the possible negative outcomes:

*I mean, as I said earlier if we go back to* tevekkül, *I link it to that*. (*…*) *this is my fate, this is how it is written, this is how I think about that*. Hacer.

**Subtheme 3d** **: Being Stronger While Knowing It isn't Always Possible**. Participants shared their reflections on their difficulties, and how these contemplations helped them to change their perspectives and consequently eased their pain. Unlike the previous sub‐theme ‘Pouring grief into Allah’, these reflections were not necessarily faith‐driven.

One of these concepts was acceptance. This included accepting one's life as it was and accepting one's limitations.

*Zeynep* talked about changing her expectations and shaping them based on the reality she was in. *Zehra*, *Hacer, Şerife* and *Ali* acknowledged their own limitations and capabilities. *Ali* cherished his ability to cope, but he also recognised that he could not be strong all the time.

Another concept was around the passage of time and the transience of life. *Yeşim* and *Neriman* stated that their troubles had diminished with time:

*They say time is the best medicine, this is actually true; time* is *the best medicine. You forget it with time, your pain eases, fades away …* Neriman.

In a similar fashion, *Neriman*, *Orhan* and *Ali* contemplated the impermanence of the world and the fact that death comes to all. This provided them with the strength to think that their difficulties would also become just small specks in the end:

*But if the person believes this, if they have a belief like that, that people die in the end, your child, your mother, your father… like* [*how people died*] *in the past, that if we keep it in our minds that we will die as well, it eases things. I am not saying this can cease a pain like losing a child, but when you keep it in your mind, it is very powerful*. Ali.

In addition to making or trying to make peace with their difficulties through being more accepting, *Elif*, *Neriman* and *Zuhal* took a further step and created positive change thanks to the adversities they faced:

*You know, developing your personality, that road doesn't end. In fact, it is very enjoyable. Reflecting on myself, trying to improve myself was really great*. Zuhal.

Even so, *Zuhal* did not dismiss the effect of the burden that the difficulties created. Similar to *Zuhal*, *Hacer* had her suspicions over whether she had washed her hands of the difficulties permanently:

*Did I really deal with those problems, finish with them, or did I just cover them and postpone them for a while? I am not so sure about that now*. Hacer.

#### Theme 4. Keeping silent in front of professionals

This theme incorporates participants' experiences and perceptions of the health systems and Health Care Professionals (HCPs) regarding mental health. Ten of the eighteen participants reported that they would be open to a referral to mental health services in the UK. Eight stated that they would not actively seek or accept such a service in the UK; six of those eight stated they would, however, consider accessing it in Türkiye. Yet, all of them, even the ones who suggested that they would seek or accept support, expressed distrust and dissatisfaction towards health services and talked about barriers to help‐seeking.

Some participants expressed antagonistic perceptions about the mental health care system and HCPs in the UK. These seemed to be fuelled by fear and distrust towards the system and the professionals. One of the most cited reasons was the fear of social services removing their children. They perceived HCP involvement as a way to ‘keep tabs’ on Muslim immigrants and thought that it might be fuelled by racism and prejudice:

Interviewer *Then from what I understand, it feels like they are making a mountain out of a molehill?*

Mesude *It doesn't feel like it; I know this is it*.

Interviewer *That they try to intrude into your private lives?*

Mesude *Exactly*.

Yeşim *True*.

Mesude *And this is not just for home life. At school, in social life. You always get those kinds of questions or stares… This is the first thing they ask; “Do you have any problems at home, are you troubled by your husband?”*.

Additionally, participants talked about how they were not satisfied with the efficiency of the NHS, for both mental and physical health. Some had had bad experiences with mental health services in particular, and the rest thought they would be ignored if they sought it.

For instance, *Ayten* stated her dissatisfaction following a 111^5^ call, which she had made. *Ayten* was told that she had ‘depression’, and she did not like how they came to that conclusion:

*When that woman told me this, I thought “how could she decide such a thing?” I mean, let a doctor see me, I mean, it was said after my responses to questions of a* [*computer*] *system, you don't know me at all* (*…*) *They completely ignored my daily struggles and told me that I was depressed*. Ayten.

*Ayten* also clarified that she was not against the idea of her being depressed, but that she did not approve of how this diagnosis was made.

*Neriman* talked about how she was prescribed psychotropic medications by her GP for psychological difficulties she was experiencing due to a long‐term separation from her three‐year‐old child:

*But after taking them* [medication], *I wasn't feeling well at all. I felt like in a dream‐world, I felt like a ghost. I was neither asleep nor awake. Then I stopped taking them but the GP told me not to stop, he said “Your body needs to adjust to the medication…*.*I mean the* [*real*] *thing* [*solution*] *is to take care of the problem* [*reuniting her with her child*]. *If I can't take care of the problem, I will be bound to take those for the rest of my life*. Neriman.

On the other hand, *Orhan* stated that he found *Lustral* 6 helpful to stop his intrusive thoughts, but due to side effects, he, too, had stopped.

The rest of the sample were not keen on taking medication for mental health. However, they explained that they were open to the idea of getting talking therapies as long as it would meet their needs. In particular, people who had difficulties with their spouses seemed open to the idea of family therapy:

*But I would like to get family therapy, for the life [experiences] to help to consolidate with the spouse, with the family, to consolidate character*. Ali.

However, participants had reservations about how to communicate with a non‐Turkish‐speaking therapist. Some stated that they would choose to have online therapy with a Turkish‐speaker over in‐person intervention with an interpreter present. Even the proficient users of English thought that having a Turkish therapist would be better. The desire to communicate in Turkish was not just related to the overt use of the language; it was also about nuances, phrases and a shared understanding embedded in the culture which could be lost in translation:

*It would be better if it is Turkish… And since we are a different community as Turks* [laughs] *we expect that they* [*Turkish therapists*] *would understand us better. Let's say it is an English person* [laughs], *if I tell them that an ox is sitting on me, an English doctor wouldn't understand that* [laughs], *but a Turkish one would*. Melike.

## DISCUSSION

The present study aimed to investigate how Turkish‐speaking immigrants in the UK experienced, understood, expressed and coped with common mental health conditions and what their relationship with help looked like. The findings suggested that the participants experienced and expressed CMHDs using emotional and physical terms. Their understanding of why they developed CMHDs was mainly psychosocial, with some biological and traditional elements. Their coping included personal, social and religious / spiritual activities as well as reflection and resilience. Most participants expressed being open to getting help from formal healthcare settings and a desire to work with Turkish‐speaking therapists; however, all expressed some distrust and dissatisfaction with the current state of the NHS.

### Symptom presentation and used expressions

All participants gave detailed accounts of their emotional distress, explicitly using emotional words. Most participants also talked about the physical presentation of their distress. Some made explicit links between their physical symptoms and emotional states. These findings replicated the results of two systematic reviews, which collected data from Turkish immigrants living in European countries and the US (Akan et al., 2023; Sempértegui et al., 2019).

A notable feature of this study was the rich, metaphorical Turkish language that participants used to describe distress, such as “an ox sitting on my chest” or “bones and heart entangling with each other.” These vivid idioms may indicate that Turkish‐speaking immigrants are not emotionally inarticulate but rather draw on culturally specific resources to express psychological pain. Such expressions carry both emotional and bodily connotations, bridging the psychological and the somatic. These findings contradict the stereotypical somatisation theory, that Turkish‐speaking people “hardly relate bodily symptoms to psychoemotional factors” (Balkir, 2013, p. 237) but “express [emotions] through allegories of bodily sensations” (Nickel et al., 2006, p. 508). The difference in popular opinion and empirical evidence reported here may stem from overgeneralisations, confounding variables, problems with translation and strategically ignoring the evidence to fit in orientalist racist and / or elitist concepts of somatisation being evaluated as a sign of deficiency – an *inability* to recognise emotions (Akan et al., 2023), despite data repeatedly showing that experiencing physical symptoms of mental health difficulties is a universal phenomenon (Haftgoli et al., 2010; Haroz et al., 2017; Simon et al., 1999; Tylee & Gandhi, 2005; Vaccarino et al., 2008).

### Explanatory models and causal attributions

Participants' accounts contained a huge variety of possible reasons for developing CMHCs. Their understandings were mainly psychosocial as they were linked to adverse life circumstances, which is in line with the findings of the recent systematic review (Akan et al., 2023) and with Reich et al.'s (2015) finding that Turkish‐speaking immigrants adopted a wider collection of causal attributions. Additionally, their understandings fluctuated uniquely in a dynamic nature within their personal narratives.

Some interviewees made connections between childhood experiences and psychological difficulties later in life, which is a universally researched phenomenon in psychological and social understandings of mental health difficulties (Herzog & Schmahl, 2018; Hughes et al., 2017). This finding also partially supports that of a previous study conducted in Germany with Turkish‐speaking immigrants, in which 17% of the Turkish‐speaking respondents suggested ‘childhood/upbringing’ without being prompted when asked about causes of psychological problems (Vardar et al., 2012).

Major traumatic life events were another suggested cause of CMHCs by the participants. The majority of the participants had had a major traumatic incident and linked it to their distress. This finding reflects previous research conducted with Turkish‐speaking immigrants (Bäärnhielm & Ekblad, 2000; Borra, 2011; Leavey et al., 2007) and other populations (Furnham et al., 2016; Read et al., 2005), which may indicate an overall acceptance of the link between trauma and psychological difficulties by diverse communities.

All participants also talked about chronic adverse circumstances influencing their mental wellbeing, a link discussed in a previous systematic review (Akan et al., 2023). On the other hand, some adverse circumstances were linked to being immigrants. The experience of being an immigrant seems to be long‐lasting. Franz et al. (2014) suggested that, even after living nearly 30 years in Germany, Turkish‐speaking participants had lower socio‐economic status, earned less and received more psychiatric diagnoses than native Germans.

Additionally, all participants agreed on the impact of relationship conflicts on CMHCs. This finding replicates previous findings for Turkish‐speakers (Balkir Neftçi & Barnow, 2016; Karanci, 1993; Vardar et al., 2012), and for general populations by systems theorists (Dallos & Draper, 2010).

A unique finding of this study was participants' thoughts and reflections on the link between their distress and their personal characteristics. These thoughts frequently came across as self‐deprecating and involved a sense of hopelessness. This is an important finding as it contradicts the literature that claims that people of the Global South locate the causes of psychological problems only in interpersonal disagreements and minimally attribute them (or do not attribute them at all) to their own personhood and actions (Marsella, 2003).

Some links between mental health and biological causes were also made by the participants. This finding mirrors previous research, which found that some Turkish‐speaking immigrants attributed their ill mental health to biological causes (Bäärnhielm & Ekblad, 2000; Leavey et al., 2007; Vardar et al., 2012).

#### Coping

The participants reported using various personal, interpersonal, community and religious activities, along with reflection and resilience, to cope with their difficulties—some of which partially aligned with previous literature (Akan et al., 2023; Mahintorabi et al., 2017; Schoenmakers et al., 2017).

Furthermore, this study exclusively highlighted that the participants did not see psychological wellness as a static/non‐changing state, but instead they adopted a dynamic model of psychological distress and wellness, acknowledging there would be good days and bad days, and their coping style reflected that.

For this population, traditional resources such as using amulets or visiting *hoca*s to undo black magic have sometimes been suggested as the primary coping mechanism for mental health difficulties (Balkir, 2013). In this sample, one participant reported using a traditional treatment (washing with vinegar water). They were also the only ones to link their distress to a traditional understanding, which might explain why they adopted a traditional intervention. This is similar to previous findings, in which Turkish‐speaking immigrants attributed their mental health difficulties to psychosocial and medical causes more than traditional ones, expressed scepticism towards traditional interventions, but still, there was some who implemented them (Akan et al., 2023; Gilgen et al., 2005; Minas et al., 2007). Coping strategies also showed intra‐group diversity. Alongside religious practices such as *sabır* (resilience), *şükür* (gratitude) and *tevekkül* (trust in God), many participants drew on reflections of acceptance, impermanence and personal growth. These strategies echo findings in other Muslim populations but also resemble acceptance‐based coping styles observed in other groups.

#### Help‐seeking attitudes

The findings indicated that most of the sample considered getting professional help as a legitimate resource. This mirrored the findings of some previous studies (Gilgen et al., 2005; Kamperman et al., 2007; Knipscheer & Kleber, 2005; Latif, 2009; Vardar et al., 2012), even though a study in Germany found motivation for psychotherapy to be lower for Turkish immigrants in comparison to the native Germans (Reich et al., 2015).

Despite wanting to seek help, all of the participants in the current study expressed distrust and dissatisfaction towards mental health help offered by UK services due to perceived racism and Islamophobia. The present study's exclusion criteria meant that none of the participants had received talking therapies, although a minority had had brief encounters with HCPs regarding mental health at the primary care level. Those who had had encounters with mental health services felt misunderstood by the professionals. Negative attitudes towards professionals (Flink et al., 2013, 2014; Latif, 2009) and discriminatory behaviour by HCPs driven by prejudice and racism (Bäärnhielm, 2004; Schinkel et al., 2019) were previously reported as barriers to seeking mental health help in this population. Although feeling misunderstood by mental health professionals is not unique to this population (Gaillard et al., 2009), the participants in this study felt this barrier could only be overcome by working with Turkish‐speaking professionals.

Their strong preference reflected not only linguistic needs but also cultural nuance and shared understanding embedded in idioms and metaphors, which they feared would be lost in translation. This preference contrasts with findings from Dutch and UK studies, where competence and empathy outweighed ethnic or language similarity (Knipscheer & Kleber, 2004; Latif, 2009). Methodological differences may partly explain this: the present study's interviews were conducted in Turkish by a partially insider researcher, which may have encouraged disclosure and a stronger emphasis on cultural fit.

In summary, the results of this study and previous literature suggest that, in theory, Turkish‐speaking immigrants are open to receiving mental health support from professionals. However, in practice, they might need to be reassured, due to their fears regarding social services and given a space to be listened to and understood.

### Strengths and limitations

This study has several strengths. It recruited participants with lived experience from across England, included both women and men and incorporated participants outside London. This way, it captured more diverse perspectives within the UK Turkish‐speaking immigrants. The use of everyday, non‐medical Turkish language embedded in the recruitment strategy allowed individuals who may otherwise have felt excluded by medical terminology to participate. Conducting interviews in Turkish with a Turkish‐speaking researcher enabled culturally nuanced, natural conversations, while analysing the data in Turkish minimised translation problems. These methodological choices are particularly valuable given that previous research with this population predominantly has relied on interpreters, which may have prevented disclosure and nuances in language. Reflexivity was also embedded throughout the process: the first author's position as a partial insider facilitated rapport, while critical reflection and team supervision helped research rigour.

Nevertheless, the study has some limitations. The selected quotations were only translated by the first author, who is a native Turkish speaker with professional‐level English skills, and quality control of the English versions was made by the second and third authors. Not involving another Turkish English speaker might be considered a limitation. The sample included only two male participants, which limited the exploration of men's experiences. Member‐checking was not formally conducted, which may have reduced opportunities for participants to clarify interpretations, although steps were taken during interviews to summarise and confirm key points. Additionally, while the sample was diverse in ethnicity (Turkish, Kurdish, Zaza) and geography, it cannot claim to represent the full breadth of the 6.5 million Turkish‐speaking immigrants worldwide. Finally, even though the group interview format offered potential benefits, such as shared reflection and collective validation, the shorter duration of this session (35 min in total) meant that the time allocated to each of the four participants was reduced, which may have impacted their contributions. For example, none of these participants discussed their childhood circumstances (please see Table 4 Theme and Sub‐Theme Distributions among Participants). This omission could be attributed to time constraints or potential group dynamics, such as social desirability and inhibition.

### Implications and recommendations

This study has several important implications for theory, policy, clinical practice, and future research.

First, the findings show that Turkish‐speaking immigrants experience and express mental health difficulties through both emotional and physical terms, accompanied by culturally rich idioms. This underscores the need for healthcare professionals to move beyond the reductive ‘somatisation’ stereotype and instead normalise physical manifestations of CMHCs. Culturally adapted interventions incorporating Turkish idioms (Eylem et al., 2015), therapies that integrate body‐focused approaches (e.g., EMDR, somatic therapies) alongside cognitive and relational interventions may be especially beneficial (Perry et al., 2019).

Second, participants highlighted the significance of social determinants — including housing, financial strain, family separation, discrimination and migration stressors — in the onset and maintenance of distress. These findings indicate that improving mental health care for this population requires structural interventions as much as clinical ones. Policymakers should prioritise inclusive housing, fairer benefits systems, accessible community spaces outside alcohol‐centred environments and stronger anti‐racism practices in public institutions, as suggested by the participants. Such recommendations echo findings from other immigrant and minority populations worldwide, suggesting that structural inequality remains a key driver of mental health disparities across contexts.

Third, the strong emphasis participants placed on family and community relationships highlights the potential of relational interventions. Group‐based and family therapies, as well as community‐based programmes, may resonate particularly well with Turkish‐speaking immigrants. At the same time, the study found considerable within‐group diversity: participants also relied on highly personal and reflective strategies, such as self‐reflection, acceptance and personal growth. This suggests that a “one‐size‐fits‐all” approach should be avoided. Clinicians should employ a person‐centred stance that recognises the coexistence of family‐ and community‐based values with individualised coping strategies.

Fourth, attitudes towards professional help revealed a complex duality: participants were generally open to receiving therapy but also deeply distrustful of UK mental health services, particularly due to fears of racism, Islamophobia and child removal by social services. These UK‐specific barriers require attention. Building trust will involve transparent communication, reassurance about confidentiality and cultural humility on the part of professionals. Establishing clear anti‐discrimination policies and accountability mechanisms in health and social care could reduce these fears and enhance engagement.

Fifth, language and culture emerged as central in shaping therapy preferences. The participants' desire to work with Turkish‐speaking professionals extended beyond language fluency, reflecting the importance of shared cultural nuance and idiomatic understanding. This differs from some findings, where competence and empathy were valued more than ethnic similarity. For practice, this may require a dual strategy: training and employing bilingual Turkish‐speaking clinicians in areas with high immigration density, while also strengthening interpreter training to ensure confidentiality and cultural nuance are preserved in areas where bilingual therapists are unavailable. Digital and online therapy platforms may also play a key role in expanding access.

Finally, while this research focused on Turkish‐speaking immigrants in the UK, the findings may echo broader patterns reported in other immigrant and ethnic minority populations’ conclusions (Harvey, 2021). Specifically, the dynamic interplay of psychosocial distress, somatic expressions and faith‐based coping strategies observed in this sample may indicate a potential for transferability to other Muslim and Global South immigrant populations, who navigate similar intersectional challenges of migration, discrimination and acculturation (Mahintorabi et al., 2017; Sulaiman‐Hill & Thompson, 2012; van Bergen et al., 2010). At the same time, this study adds unique insights, including the frequent self‐reflection, metaphor‐rich language of distress, intra‐community tensions and the specific fear of state intervention in personal lives. Future research should further explore how these unique and shared elements interact across cultural groups, in order to build a more nuanced and inclusive understanding of immigrant mental health.

## CONCLUSION

Taken together, these findings challenge simplistic stereotypes of Turkish‐speaking immigrants as “somatising” as an alternative to emotion identification and expression. The hybrid explanatory models and causal attributions observed in this sample challenge binary distinctions between Global North “psychologisation” and Global South “somatisation.” Instead, they suggest a dynamic and fluid model of mental health, characterised by a varied selection of coping resources and complex attitudes towards seeking help among Turkish‐speaking immigrants. These findings are likely to echo future findings in other Global South populations.

## AUTHOR CONTRIBUTIONS

**Ayse Akan:** Conceptualization; investigation; writing – original draft; methodology; writing – review and editing; formal analysis; data curation; project administration. **Gill Green:** Methodology; writing – review and editing; supervision. **Frances Blumenfeld:** Writing – review and editing; supervision; methodology. **Danny Taggart:** Conceptualization; writing – review and editing; methodology; supervision. **Susan McPherson:** Conceptualization; writing – review and editing; supervision.

## FUNDING INFORMATION

AA's doctoral studies were funded by the Turkish Ministry of National Education (*Milli Eğitim Bakanlığı*) under the 1416 scholarship programme. AA also used funds from the University of Essex for her doctoral research. There were no financial contributions from the Turkish Ministry of National Education and the University of Essex during the preparation and publication of this paper. Open access fees were covered through the ANKOS agreement.

## CONFLICT OF INTEREST STATEMENT

The authors declare no conflict of interest.

## ETHICS STATEMENT

Ethical approval from the University of Essex was obtained, and the ethical requirements were followed.

## CONSENT

Informed verbal and written consent was obtained from the participants.

## Data Availability

Data is not made available due to the sensitive nature of qualitative data.

## Footnotes

^1^
In this paper, we use the term common mental health *conditions* rather than *disorders* in order to be consistent with and reflect the most recent UK Adult Psychiatric Morbidity survey terminology (NHS England, 2025).

^2^
In this context, the *Global North* refers to wealthy, industrialised nations primarily in North America and Europe that define modern psychological standards, while the *Global South* encompasses developing nations in Africa, Latin America and Asia with diverse cultural frameworks. The distinction is less about geography and more about the historical power imbalance between former colonisers and colonised regions, particularly regarding how mental health is defined and treated.

^3^
Türkiyeli means ‘from Türkiye’ and is generally used by non‐Turkic populations from Türkiye.

^4^
An Islamic ritual involving bodily cleaning rituals, physical movements and saying verbal prayers, and had started practising that regularly.

^5^
This NHS service offers the callers advice on the symptoms which they are having with the help of a computerised system and makes suggestions to callers, such as going to A&E or visiting their GP.

^6^
The brand name for sertraline, which is a selective serotonin re‐uptake inhibitor (SSRI) class anti‐depressant.
